# Transcriptome Analysis of the *Clostridioides difficile* Response to Different Doses of *Bifidobacterium breve*

**DOI:** 10.3389/fmicb.2020.01863

**Published:** 2020-07-31

**Authors:** Jingpeng Yang, Hong Yang

**Affiliations:** State Key Laboratory of Microbial Metabolism, School of Life Sciences and Biotechnology, Shanghai Jiao Tong University, Shanghai, China

**Keywords:** *Bifidobacterium breve*, *Clostridioides difficile*, transcriptomic analysis, dosage, toxin production

## Abstract

Probiotics are widely used in the prevention of *Clostridioides difficile* infection (CDI). The precise dosage of probiotics is a challenge. In this study, *Clostridioides difficile* ATCC 9689 (CD) was exposed to different doses of *Bifidobacterium breve* (YH68). A transcriptomic analysis was performed on CD cells that were separately exposed to low or high doses of YH68 cell-free culture supernatant (CFCS; CDL; or CDH, respectively). The results showed that the inhibitory effect of YH68 (cell pellets or CFCS) on the growth and the damage to the cell membrane integrity of CD exhibited a dose-response relationship at the physiological level. At the transcriptional level, a large number of differentially expressed genes (DEGs) were concentrated in amino acid, carbohydrate, energy metabolism and membrane transport in CDL and CDH cells, suggesting that both doses of YH68-CFCS triggered a significant change in activities in these metabolic pathways. Importantly, a significant stimulation or suppression was found in the pathogenic pathways (quorum sensing, signal transduction, flagellar assembly, biofilm formation, and drug resistance) of CDL and CDH cells, whereas there were some differences between the two doses. For example, the expression levels of genes related to quorum sensing and signal transduction in CDH cells were suppressed significantly, whereas genes encoding toxin production and sporulation factors were enhanced; in CDL cells, the expression levels of genes associated with flagellar assembly and biofilm formation were suppressed, whereas genes associated with drug resistance were upregulated significantly. These results indicated that the inhibitory effect of YH68-CFCS against CD, especially in pathogenic and metabolic aspects, did not demonstrate a dose-response relationship at the transcriptional level.

## Introduction

*Clostridioides difficile* is a Gram positive obligate anaerobic bacteria that can produce spores (Gil et al., 2017). Infectious diarrhea induced by *C. difficile* (CDI) accounts for nearly 25% of antibiotic-associated diarrhea (AAD), and this disease imparts substantial health and financial burdens on society (Larcombe et al., 2016). Currently, antibiotics remain the mainstay of CDI therapy despite having many side effects, such as destruction of the intestinal microbiota and immune system (Palleja et al., 2018), and emergence of multidrug-resistant strains (Aptekorz et al., 2017). In recent years, probiotics have often been used in the treatment of various clinical diseases due to their outstanding antibacterial activity with very few side effects (Rondanelli et al., 2017). Meanwhile, a growing body of studies has shown that probiotics not only protect or rebuild the normal microbial community structure in the gut but also activate the immune system (Goldenberg et al., 2018). The application of probiotics in the food field has a wide historical span (Plaza-Diaz et al., 2019), and thus, their safety is widely recognized by the public. Even in recent decades, probiotics have frequently been used as adjuvant agents in clinical treatment (Sanders et al., 2019), and their high safety in the public mind has remained unchanged. A report from Shen et al. (2017) revealed that the therapeutic effect of probiotics or probiotics in combination with antibiotics on CDI or rCDI has significant efficacy, whereas the precise dosage of probiotics is a challenge. To date, the dosage of probiotics used in the prevention and treatment of CDI is not clear. Many physicians believe that a higher probiotic dosage used results in a better therapeutic effect, although there is no theoretical basis to support this attitude. The most probable reason behind this trust is that probiotics are not traditional medicines (such as antibiotics), and most of them have exerted no side effects in the vast majority of clinical CDI treatments. Interestingly, a few studies have demonstrated that probiotics might interfere with the normal microbial community structure in the gut, especially when the density of probiotics reaches a high level (Langella and Chatel, 2019). A previous study revealed that 10^10^ CFU of probiotics induced a transient structural change in the gut microflora and impacted host immune and endocrine function (Derrien and van Hylckama Vlieg, 2015); however, no further studies uncovered whether this change had a significant impact on human health. Considering the demand and development of precision medicine, additional attention should be focused on the precise dosage of personalized probiotic strains used in the treatment of specific diseases (Suez et al., 2019), such as CDI.

To our knowledge, there is no study determining whether probiotics and their metabolites at different doses can have the same or different effects on *C. difficile*, especially on changes at the transcriptional level. Data from our previous studies indicated that the antibacterial activity of *Bifidobacterium breve* YH68 against *C. difficile* ATCC 9689 (CD) is significant (Yang and Yang, 2018, 2019a). In this study, we used several physiological indexes in combination with a transcriptome analysis to explore the changes in CD cell responses to YH68 cell pellets or cell-free culture supernatant (CFCS) at different doses, especially focusing on the transcriptional differences in CD cells triggered by low and high doses of YH68-CFCS.

## Materials and Methods

### Strains and Growth Conditions

*Clostridioides difficile* ATCC 9689 (CD) was purchased from the American Type Culture Collection, and *B. breve* YH68 (CGMCC No. 14096) was provided by Jiaxing Innocul – Probiotics Co., Ltd. (Jiaxing, China). CD and YH68 were individually cultured anaerobically in brain heart infusion (BHI) broth and de Man Rogosa Sharpe medium supplemented with 0.05% (w/v) L-cysteine (MRSC) broth for 24–48 h at 37°C (AnaeroGenTM, Oxoid Ltd., Basingstoke, United Kingdom).

### Preparation of Different Doses of YH68

YH68 was inoculated into 50 mL fresh MRSC broth and cultured anaerobically to logarithmic phase at 37°C. Then, the bacterial solution of YH68 was individually diluted to 10^3^, 10^5^, 10^7^, and 10^9^ CFU/mL with sterile water, as four dosages groups. After that, CFCS and cell pellets (cell) from these four dosages groups were separately collected after centrifugation (4°C, 12,000 r/min, and 10 min) and sterile filtration (0.22 μm); the cells of YH68 were resuspended in sterile water, and finally all these YH68 CFCS and cell suspension from four dosages groups reached a same volume (5 mL). Each dosage group was numbered as “dose index-YH68,” including two treatments (YH68-CFCS and YH68-cell), e.g., 10^3^ CFU/mL group refers to 3-YH68-CFCS and 3-YH68-cell.

### Preventive and Therapeutic Inhibition

Clinically, probiotics are often used to prevent *C. difficile* invasion (preventive inhibition) or alleviate the intestinal tissue damage caused by *C. difficile* (therapeutic inhibition). CD was inoculated anaerobically into 50 mL fresh BHI broth for 48 h at 37°C. After centrifugation (4°C, 12,000 r/min, and 10 min), cell pellets were collected from the CD bacterial solution, resuspended in the sterile water and adjusted to 10^8^ CFU/mL.

One mL of YH68-cell or YH68-CFCS from each dosage group was, respectively, inoculated into 8 mL fresh BHI broth and cultured for 2 h at 37°C before 1 mL of CD bacterial suspension (10^8^ CFU/mL) was added. Four dosages groups contained a total of eight treatments (YH68-cell and YH68-CFCS). The mixed bacterial solution was cultured anaerobically for 24 h at 37°C, and then 200 μL samples were removed from each group, inoculated onto *Clostridioides difficile* moxalactam norfloxacin agar (CDMN) selective medium (Oxoid Ltd., Basingstoke, United Kingdom), and anaerobically cultured for 48 h at 37°C to determine viable CFU/mL. The control group used 1 mL of sterile water for replacement. This assay was repeated three times independently.

$$\mathrm{Preventive}\mathrm{inhibition}\mathrm{rate}\left(\%\right)=\left(1-\mathrm{CD}\mathrm{in}\mathrm{different}{\mathrm{groups}}_{24\text{h}}/\mathrm{CD}\mathrm{in}{\mathrm{control}}_{24h}\right)\times 100\%$$

One mL of CD bacterial suspension (10^8^ CFU/mL) was first inoculated into 8 mL fresh BHI broth and anaerobically cultured for 2 h at 37°C. Then, 1 mL of YH68-CFCS or YH68-cell from four dosages groups was, respectively, added. The following steps are the same as those for preventive inhibition. This assay was repeated three times independently.

Therapeuticinhibitionrate(%)

$$=\left(1-\mathrm{CD}\mathrm{in}\mathrm{different}{\mathrm{groups}}_{24\text{h}}/\mathrm{CD}\mathrm{in}{\mathrm{control}}_{24\text{h}}\right)\times 100\%$$

### Toxin Production and *tcdA/tcdB* Gene Expression

Ten mL CD (10^8^ CFU/mL) was inoculated into 190 mL fresh BHI broth and anaerobically cultured for 48 h at 37°C. Then, the CFCS of CD was collected by centrifugation (4°C, 12,000 r/min, and 10 min) and filtration (0.22 μm). All these CD-CFCS was divided into nine equal parts (each part was 9 mL), following with 1 mL of YH68-cell, YH68-CFCS from four dosages groups (1 mL sterile water as the control) was added. Subsequently, all these groups were anaerobically cultured at 37°C. 200 μL of samples was removed from each group at 24 and 48 h, following which the CFCSs of samples from all these groups were filter sterilized (0.22 μm) to assess soluble TcdA/B levels using a *C. difficile* Toxin A/B II ELISA kit (Runyu Ltd, Shanghai, China) according to the manufacturer’s instructions. Samples were diluted when necessary to obtain readings within the linear range of the standard (3–3000 ng/mL). All samples were tested in triplicate and this assay was repeated at least three times independently.

One mL CD (10^8^ CFU/mL) was inoculated into 99 mL fresh BHI broth and anaerobically cultured to logarithmic phase at 37°C, and the cultures were divided into five equal parts (each part was 9 mL); then, 1 mL of YH68-CFCS from four dosages groups was added before anaerobically co-cultured (1 mL sterile water as the control). Subsequently, 1 mL of samples was removed from each group at 24 and 48 h. Cell pellets were collected by centrifugation (4°C, 12,000 r/min, and 10 min) and filtration (0.22 μm). RNA was isolated from the collected CD cell pellets using an RNA-prep Pure Kit for Bacteria (TianGen Biotech, Beijing, China) according to the manufacturer’s instructions. Contaminant genomic DNA was removed by two rounds of DNase treatment (DNA-free Kit; Tiangen), and the final RNA yield and quality were assessed by ultraviolet absorbance measurement and agarose gel electrophoresis, respectively. The expression levels of the *tcdA* and *tcdB* genes were assessed by real-time qPCR using a method reported by Aldape et al., 2015, with some modifications; the method has been described below with specific primers (Persson et al., 2008). cDNA was synthesized from each sample (500 ng) using All-in-One First-Strand cDNA Synthesis SuperMix (TransGen Biotech, Beijing, China) and amplified using TransStart Top Green qPCR SuperMix (TransGen Biotech) in a Mastercycler ep realplex system (Eppendorf, Hamburg, Germany) as follows: 30 s at 94°C, followed by 45 cycles at 94°C for 5 s, 55°C for 15 s, and 72°C for 10 s. *C. difficile* 16S rRNA served as an internal control. Relative gene expression was determined using the 2^–ΔΔ*Ct*^ method. All samples were tested in triplicate and this assay was repeated at least three times independently.

### Cell Membrane Integrity

A damaged cell membrane results in the leakage of intracellular ATP and UV-absorbing materials (nucleic acid substances and proteins) from CD cells. Thus, the changes in extracellular ATP and UV-absorbing material levels accurately reflect the degree of damage to CD cells. 1 mL of a CD cell suspension (10^8^ CFU/mL) was mixed with 1 mL of different doses of YH68-CFCS before anaerobically co-cultured at 37°C. Sampling 200 μL from the co-cultured mixed bacterial solution at 3 h, and the CFCS of the mixed bacterial solution was collected after centrifugation (4°C, 12,000 r/min, and 10 min) and filtration (0.22 μm). 100 μL of the CFCS was used to determine the extracellular ATP levels by a previously described bioluminescence-based method (Suzuki et al., 2005) using a microplate reader (PE and EnSpire 2300) and a CellTiter-Glo 2.0 Kit (Promega, United States). The rest 100 μL of the CFCS was used to determine the extracellular UV-absorbing material levels according to a method reported by Liu et al., 2017, with some modifications. The absorbance of the CFCS was recorded at OD_260_ and OD_280_ using a UV spectrophotometer (Lamda 950, China). All samples were tested in triplicate and this assay was repeated at least three times independently.

### Preparation of Sequencing Libraries for RNA-Seq

According to the physiological results, we are interested in whether there are same, or different changes at the transcriptome level of CD cells that were separately exposed to the different doses of YH68-CFCS, especially CD cells that were exposed to low or high doses of YH68-CFCS (CDL or CDH, respectively). 50 mL of CD (10^8^ CFU/mL) was inoculated into 2000 mL fresh BHI broth and anaerobically cultured for 48 h at 37°C; the cell pellets from CD bacterial solution were collected by centrifugation (4°C, 12,000 r/min, and 10 min), filtration (0.22 μm), concentration and resuspended in 300 mL fresh BHI broth. Then, the CD cell suspension was divided into three equal parts (each part was 100 mL), and two parts of them were separately mixed with 50 mL of 5-YH68-CFCS (low dosage) and 9-YH68-CFCS (high dosage). The rest part without YH68-CFCS was regarded as the control (YH68-CFCS was replaced by 50 mL fresh MRSC broth). The three different treatments of CD cells were renamed CDL, CDH, and CDC. All these groups were anaerobically cultured for 3 h at 37°C. Subsequently, total RNA was extracted from CDC, CDL, and CDH cells using an RNA-prep Pure Kit for Bacteria (TianGen Biotech, Beijing, China). RNA quality and quantity were monitored by agarose gel electrophoresis, NanoDrop 2000 spectrophotometer (Nanodrop Technologies, Thermo Fisher, United States), and Agilent 2100 (Agilent Technologies, Santa Clara, CA, United States) instruments. rRNA was removed from the total RNA by a Ribo-Zero rRNA Removal Kit (Bacteria). The mRNA was purified and fragmented by chemical reagents at high temperatures, and these fragments were used as templates to synthesize the first-strand cDNA in the presence of random primers (SuperScript II, Invitrogen, Carlsbad, CA, United States). “dTTP” was replaced by “dUTP” during second-strand cDNA synthesis, which was performed using DNA polymerase I and RNase H before end-repair, dA tailing and adapter ligation. The adapter-modified fragments were then purified and amplified to create the final cDNA library. After that, the library was quantified by the Pico Green and fluorescence spectrophotometer method (Quantifluor-ST fluorometer, Promega, E6090; Quant-iT PicoGreen dsDNA Assay Kit, Invitrogen, P7589). cDNA sequencing was conducted on an Illumina HiSeq platform based on synthesis technology. The part of different treatment in this assay was repeated at least ten times independently. After every independent repeat assay, RNA quality and quantity of CD cells were monitored. Finally, nine samples with the high-quality RNA from CDC, CDL, and CDH were selected to build RNA-seq libraries.

### Bioinformatic Analysis

Clean data were obtained from the filtered raw sequence data (FASTQ format) using the Illumina HiSeq built-in software and mapped onto the annotated *C. difficile* strain ATCC 9689 reference genome in NCBI (GCA_001077535.2_ASM107753v2_genomic.fna). Then, the CDC, CDL and CDH RNA-seq maps were generated through Bowtie2^1^. HTSeq 0.6.1 p2^2^ was used to evaluate the fragments per kilobase of exon per million fragments mapped (FPKM) for all genes. Differentially expressed genes (DEGs) of the three groups were identified by DESeq (version 1.18.0). The false discovery rate (FDR) was calculated to correct the *p*-value obtained from independent statistical hypothesis testing. DEGs were identified as genes with a *P*-value < 0.05 and log2| fold change| > 1. The ggplots2 and pheatmap packages of R software were used to create DEG volcano and cluster plots, respectively. GO enrichment analysis was implemented to distribute DEGs with topGO. The ClueGO plugin of Cytoscape (3.7.2) was used to further visually demonstrate DEG relationships in the top 10 GO terms (Bindea et al., 2009, 2013). Kyoto Encyclopedia of Genes and Genomes (KEGG) enrichment analysis was performed to identify the major categories of DEGs based on molecular-level information.

### RT-qPCR Validation

An RT-qPCR assay was performed on CDC, CDL and CDH cells with the same method used in the *tcdA/tcdB* gene expression analysis. Eight DEGs (up- and downregulated) from different pathways in CDL and CDH cells were selected to confirm the RNA-Seq results using the primers listed in Supplementary Table S1. The log2 fold change was calculated after normalization to the *C. difficile* 16S rRNA internal control.

### Statistical Analysis

The differences between the groups were examined by the one-way analysis of variance (ANOVA) followed by Dunnett’s multiple comparisons test using Minitab 16.2.3 software (Minitab Inc., State College, PA, United States). A *P*-value of <0.05 was considered statistically significant.

## Results

### Preventive and Therapeutic Inhibition

**FIGURE 1:**
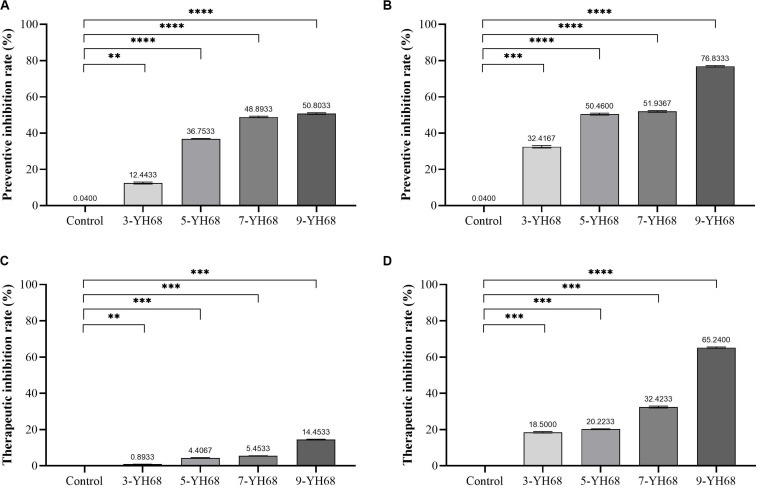
Inhibitory effects of YH68 at different doses against *C. difficile* growth. **(A)** Preventive inhibition rate of YH68-cell, **(B)** Preventive inhibition rate of YH68-CFCS, **(C)** Therapeutic inhibition rate of YH68-cell, **(D)** Therapeutic inhibition rate of YH68-CFCS. (Sterile water is used as a control, *^∗∗^P* < 0.01, *^∗∗∗^P* < 0.001, and ^****^P < 0.0001; Dunnett’s multiple comparisons test).

The results from the preventive and therapeutic inhibition simulation showed that the growth of CD was inhibited by both cells and CFCS of YH68 (Figure 1). Moreover, the inhibitory effect against CD became stronger as the dose of YH68 increased. In the test of preventive inhibition, with the increased doses of YH68-cell (and YH68-CFCS), the inhibitory rates reached 12.44% (32.42%), 36.75% (50.46%), 48.89% (51.94%), and 50.80% (76.83%), respectively. For therapeutic inhibition, the inhibitory rates of YH68-cell (and YH68-CFCS) against CD reached 0.89% (18.50%), 4.41% (20.22%), 5.45% (32.42%), and 14.45% (65.24%), respectively. The antibacterial activity of YH68-CFCS against CD was stronger than that of YH68-cell. Overall, with regard to prevention, both YH68-cell and YH68-CFCS exerted a prominent preventive effect, and all the inhibitory rates of YH68 at different doses against CD reached more than 50%. However, the inhibitory rates of YH68-cell at different doses against CD were not prominent for the therapeutic inhibition aspect, including the highest dose of YH68-cell (14.45%); meanwhile, the inhibitory effect of YH68-CFCS was relatively outstanding, although the inhibitory rates of YH68-CFCS at different doses against CD were lower than 50%, except for that of 9-YH68-CFCS (65.24%).

### Toxin Production and the Expression Levels of *tcdA/tcdB*

**FIGURE 2:**
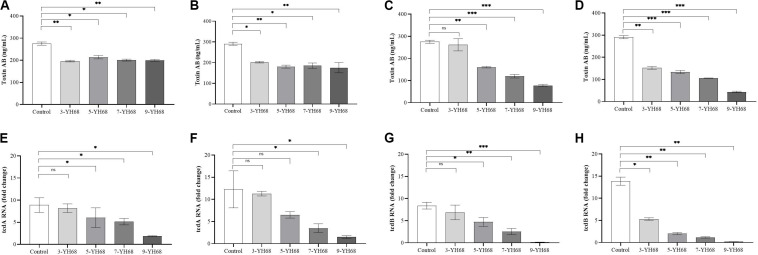
Inhibitory effects of YH68 at different doses against toxin production and virulence gene expression of *C. difficile*. **(A)** Inhibitory effects of YH68-cell against toxin production at 24 h, **(B)** Inhibitory effects of YH68-cell against toxin production at 48 h, **(C)** Inhibitory effects of YH68-CFCS against toxin production at 24 h, **(D)** Inhibitory effects of YH68-CFCS against toxin production at 48 h, **(E)** Inhibitory effects of YH68-CFCS against *tcdA* gene expression at 24 h, **(F)** Inhibitory effects of YH68-CFCS against *tcdA* gene expression at 48 h, **(G)** Inhibitory effects of YH68-CFCS against *tcdB* gene expression at 24 h, and **(H)** Inhibitory effects of YH68-CFCS against *tcdB* gene expression at 48 h. (Sterile water is used as a control, ns, not significant, ^∗^*p* < 0.05, *^∗∗^P* < 0.01, and *^∗∗∗^P* < 0.001; Dunnett’s multiple comparisons test).

The toxin production of CD was reduced by YH68, and the effect of YH68-CFCS was stronger than that of YH68-cell (Figure 2). Compared with the control (275.45 ± 6.42 ng/mL at 24 h, 290.97 ± 6.42 ng/mL at 48 h), YH68-cell reduced some of the toxin production. However, the toxin level did not continue to decline as the dose of YH68-cell increased and finally stabilized at 200 ng/mL, suggesting that there is no significant correlation between the decrease in toxin level and the increase in YH68-cell dose. In addition, a slight decrease in the toxin level was found in all groups at 48 h compared with that at 24 h, except for 3-YH68-cell. Conversely, YH68-CFCS showed a significant dose response, and the toxin level decreased with increasing dose. The toxin levels of 3-, 5-, 7-, and 9-YH68-CFCS individually reached 261.65 ± 24.60, 160.50 ± 3.08, 119.69 ± 7.29, and 77.73 ± 4.63 ng/mL at 24 h and 151.87 ± 5.56, 133.48 ± 6.23, 105.89 ± 0.89, and 43.24 ± 3.08 ng/mL at 48 h, respectively. Overall, YH68-CFCS had a greater ability than YH68-cell to reduce CD toxins. In terms of virulence genes, the expression levels of *tcdA* and *tcdB* decreased gradually with increasing doses of YH68-CFCS. The expression levels of *tcdA* increased at 48 h (except for those of 7-YH68-CFCS and 9-YH68-CFCS) compared with those at 24 h. For *tcdB*, a significant weakened effect was found with increasing doses of YH68-CFCS, suggesting a dose-response relationship.

### Cell Membrane Integrity

**FIGURE 3:**
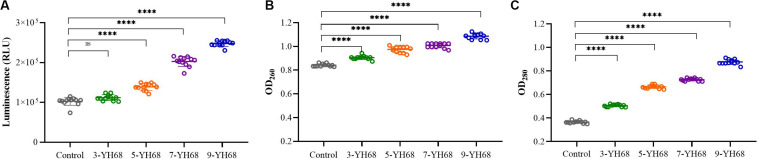
Effects of YH68-CFCS at different doses on *C. difficile* cells. **(A)** Luminescence values represent the extracellular ATP level, **(B)** OD_260_ values represent the extracellular nucleic acid substances level, **(C)** OD_280_ values represent the extracellular protein substances level. (Sterile water is used as a control, ns, not significant, and ^****^P < 0.0001; Dunnett’s multiple comparisons test).

The changing levels of ATP (luminescence value) and UV-absorbing materials (OD_260/280_ value) from CD cells were determined and regarded as indicators of cytolysis and random pore formation. The extracellular luminescence and OD_260/280_ values from CD cells increased gradually with increasing doses of YH68-CFCS (Figure 3), suggesting a serious leakage of intracellular ATP, nucleic acid and protein substances from CD cells. These results indicated that damage to CD cell membrane integrity was positively correlated with an increased dose of YH68-CFCS.

### Transcriptome Analysis

The results from the analysis of growth, toxin production, *tcdA/tcdB* expression levels and cell membrane integrity of CD indicated that the inhibitory effect of YH68-CFCS against CD gradually increased with the dose of YH68-CFCS. Is there any difference between different doses of YH68-CFCS against CD? If there is, what is the reason behind this phenomenon? Is the antibacterial activity of high dosage against CD certainly better than that of low dosage? Considering these issues, we chose 5-YH68-CFCS (low dose), and 9-YH68-CFCS (high dose) to treat CD cells. CD cells treated with these two doses of YH68-CFCS were renamed CDL and CDH, respectively; meanwhile, CD cells treated without YH68-CFCS were used as a control (CDC). A transcriptome analysis was performed on CD cells to explore the differences between CDC, CDL, and CDH cells. In the process of RNA extraction, we found that the RNA integrity number (RIN) decreased as the processing time increased, suggesting that the degradation of total RNA from CD cells was substantial over time. This phenomenon was found in groups with or without YH68-CFCS. For prokaryotic samples, RIN more than 7.0 can be accepted (RIN ≥ 7.0). We explored the change in RIN under different processing times (1, 2, 3, 6, 12, 24, 36, and 48 h) and confirmed that the RIN was acceptable within 3 h. Finally, total RNA of CDC, CDL, and CDH cells that were treated for 3 h was used for cDNA library construction.

**FIGURE 4:**
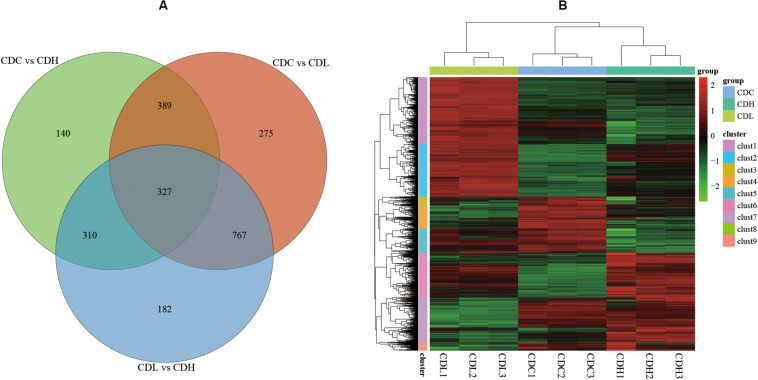
*C. difficile* transcriptome in different groups. **(A)** Venn diagram showing the differentially expressed genes that were shared or unique in CDC, CDL, and CDH. **(B)** Heat map showing clustering of differentially expressed genes in CDC, CDL, and CDH. Red parts represent high expression gene clusters, while green parts represent low expression gene clusters.

The density distribution of FPKM can be used to investigate the gene expression pattern of a sample. Generally, moderately expressed genes account for the majority, while lowly expressed and highly expressed genes account for only a small proportion (Supplementary Figures S1,S2). The density distribution of FPKM in CDC, CDL, and CDH cells exhibited a high level of similarity. PCA analysis and Venn diagram showed that CDC, CDL, and CDH are three different states (Supplementary Figure S1 and Figure 4). The DESeq package (version 1.18.0) from R software was used to analyze the differences in gene expression (log2| fold change| > 1, *P*-value < 0.05). For CDL relative to CDC (CDC vs CDL), we detected 1758 DEGs, including 867 downregulated and 891 upregulated genes. Relative to CDC, we detected 1166 DEGs in CDH (CDC vs CDH), with 536 genes decreased in expression and 630 increased. For CDH relative to CDL (CDL vs CDH), we detected 1586 DEGs, with 761 genes being decreased in expression and 825 being increased. There was significant overlap of the DEGs in CDL and CDH relative to those in CDC, while highly expressed gene groups in CDC, CDL, and CDH cells varied.

#### Global Analysis of Transcriptomic Response and RT-qPCR Validation

**FIGURE 5:**
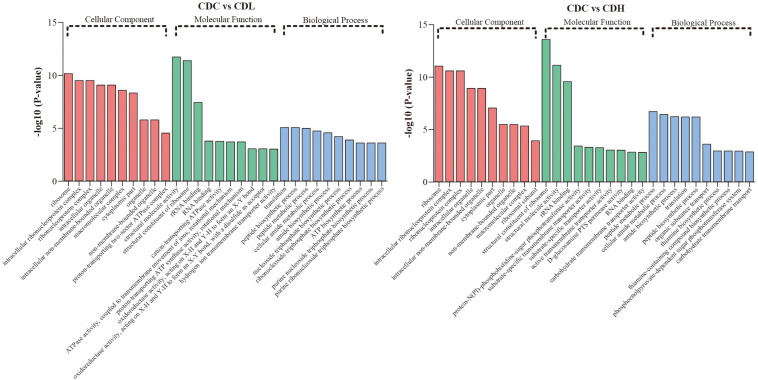
GO term analysis of *C. difficile* DEGs in different groups. Top 10 GO terms belonged to cellular component, molecular component, and biological process were, respectively, included in CDL and CDH (*P*-value < 0.05 was the significance threshold).

**FIGURE 6:**
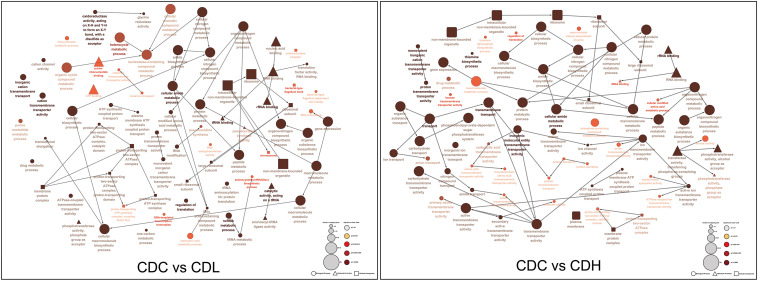
ClueGO analysis of the top 10 GO terms in *C. difficile*. “□” represent cellular component, “△” represent molecular component, and “∘” represent biological process.

Functional categories of DEGs were annotated by Gene Ontology (GO) enrichment analysis, belonging to the three categories cellular component (CC), molecular function (MF), and biological process (BP). According to the magnitude of the *P* value, the top 10 GO terms that included the most DEGs in CDL and CDH cells were individually collected and analyzed (Figure 5 and Supplementary Table S3). Within the CC category, compared with those in CDC cells, the main terms that DEGs were distributed in CDL and CDH cells were ribosomes, macromolecules, and intracellular organelles. For the MF category, the top terms by frequency in CDL and CDH cells focused on structural molecule activity, ribosome composition, and the synthesis of ribosome RNA, but with some subtle differences, reflecting the increased levels of rRNA binding, cation-transporting ATPase activity in CDL cells and of protein-N(PI)-phosphohistidine-sugar phosphotransferase activity and substrate-specific transmembrane transporter in CDH cells. With regard to the BP category, the top 5 terms in CDL and CDH cells were the same and located mostly in translation, peptide biosynthetic and metabolic process, and cellular amide metabolic process. ClueGO analysis was further performed to provide a network relation among the top 10 GO terms in the CC, MF, and BP categories for CDL and CDH cells (Figure 6). Both of CDL and CDH have the same main terms in CC category, such as ribosome, intracellular non-membrane-bounded organelle, and non-membrane-bounded organelle. For the MF category, CDL, and CDH focused on RNA and rRNA binding, but with some subtle differences, reflecting the increased levels of transferase activity, transferring phosphorus-containing groups, phosphotransferase activity, alcohol group as acceptor in CDH cells. For the BP category, the main terms in CDL and CDH were cellular amide metabolic process, protein metabolic process, peptide metabolic process, organic substance biosynthetic process, and macromolecule metabolic process, but with some subtle differences, reflecting the increased levels of transmembrane transport, transport in CDH cells. There was a tight relationship between all these main terms above in CDL and CDH cells, and these terms were significantly and aggregated a large number of genes. Overall, results of ClueGO analysis were in line with that of GO enrichment analysis and reflected the relationships of different GO terms in GO enrichment analysis.

**FIGURE 7:**
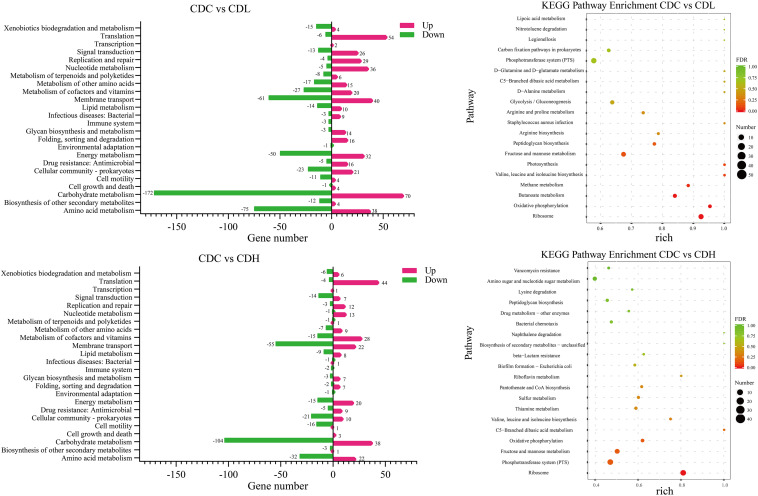
KEGG pathway analysis of the *C. difficile* DEGs in different groups. Rich (Rich factor) represent the ratio of the DEGs in the pathway to their annotated DEGs. The larger of the rich factor, the greater of enrichment degree. The size and color of solid circle, respectively, represent the number of differential genes enriched in this pathway and their FDR values. The range of FDR values is 0 to 1. The closer to zero value represents the enrichment is more significant.

**FIGURE 8:**
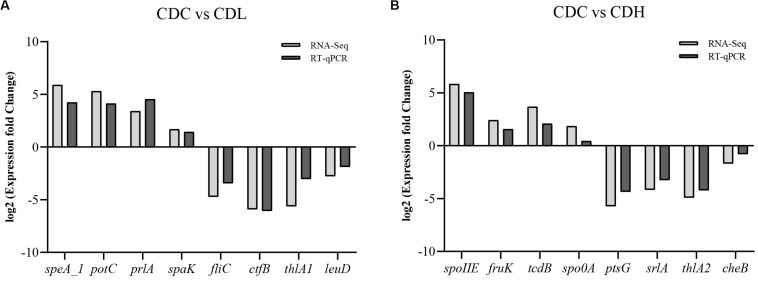
Comparison of RT-qPCR and RNA-Seq data for selected key *C. difficile* ATCC 9689 genes (the R^2^ value was 0.92 in CDL **(A)** and 0.91 in CDH **(B)** samples).

To further systematically analyze the DEG functions in CDL and CDH cells, KEGG enrichment analysis was performed to categorize DEGs into different pathways based on bioinformatic databases, including 24 functional categories (Figure 7 and Supplementary Table S4). Compared with those in CDC cells, the largest number of the top ten DEGs in CDL cells belonged to carbohydrate metabolism (242, 24.22%), amino acid metabolism (113, 11.31%), membrane transport (101, 10.11%), energy metabolism (82, 8.21%), translation (60, 6.01%), cofactor and vitamin metabolism (47, 4.70%), cellular community—prokaryotes (44, 4.40%), nucleotide metabolism (41, 4.10%), signal transduction (39, 3.90%), and replication and repair (33, 3.30%). For CDH cells, the largest number of the top ten DEGs belonged to carbohydrate metabolism (142, 24.07%), membrane transport (77, 13.05%), amino acid metabolism (54, 9.15%), translation (48, 8.14%), cofactor and vitamin metabolism (43, 7.29%), energy metabolism (35, 5.93%), cellular community—prokaryotes (31, 5.25%), signal transduction (21, 3.56%), cell motility (17, 2.88%), and lipid metabolism (17, 2.88%). Overall, most of the most enriched top ten DEG functions in CDL and CDH cells were the same. Moreover, DEGs of CDL and CDH cells were involved mainly in pathways of carbohydrate metabolism, amino acid metabolism, membrane transport, and energy metabolism. Twenty-eight and 27 DEGs (up- and downregulated) from different pathways in CDC, CDL, and CDH cells were selected, and subsequently, an RT-qPCR assay was performed on the same RNA samples to validate the RNA-Seq data. The results of RT-qPCR were generally in accordance with those of RNA-Seq, suggesting that the RNA-Seq results were valid (Figure 8).

Carbohydrate and amino acid metabolism is important for the normal growth of CD. The largest number of DEGs in CDL and CDH cells was focused on carbohydrate metabolism, including 172 downregulated and 70 upregulated genes in CDL and 104 downregulated and 38 upregulated genes in CDH cells. In terms of inhibiting carbohydrate metabolic activity, the most downregulated genes belonged to fructose and mannose metabolism, butanoate metabolism, and glycolysis/gluconeogenesis in CDL cells, whereas CDH cell changes were focused on fructose and mannose metabolism, starch and sucrose metabolism, and amino sugar and nucleotide sugar metabolism. One significant difference between CDL and CDH cells was butanoate metabolism. All 26 DEGs involved in this pathway in CDL cells were downregulated, whereas there were only 7 DEGs in CDH cells. With regard to amino acid metabolism, some top pathways were different between CDL and CDH cells. For example, the top pathways involved in this level in CDL cells were focused on alanine, aspartate and glutamate metabolism, cysteine and methionine metabolism, and arginine and proline metabolism; for CDH cells, cysteine and methionine metabolism, arginine and proline metabolism, and valine, leucine and isoleucine biosynthesis were the top pathways.

Energy metabolism and membrane transport are closely related to carbon and nitrogen metabolism, which play critical roles in the growth of CD. The top 7 pathways with the most DEGs in CDL and CDH cells were oxidative phosphorylation, carbon fixation pathways in prokaryotes, methane metabolism, photosynthesis, sulfur metabolism, carbon fixation in photosynthetic organisms, and nitrogen metabolism, especially oxidative phosphorylation, which ranked first in both CDL and CDH cells, with only a slight difference. Genes encoding V-type ATP synthase and iron-only hydrogenase in CDL cells were downregulated 3.4- to 6.7-fold and 1.4- to 2.9-fold (log2FC), respectively, and 8 genes encoding ATP synthase subunits were upregulated 1.7- to 2.6-fold. For CDH cells, there were 10 upregulated genes involved in V-type ATP synthase (1.1- to 2.2-fold) and ATP synthase subunits (1.5- to 2.0-fold), whereas genes belonging to iron-only hydrogenase were downregulated 1.1- to 1.3-fold. Methane metabolism was the second largest pathway of energy metabolism, and CDIF1296T_00895 and CDIF1296T_00896, encoding acetyl-CoA decarbonylase/synthase, in CDL cells were significantly downregulated (5.0- to 5.7-fold). In CDH cells, sulfur metabolism ranked second, and 6 genes associated with ABC transporter permease (CDIF1296T_03071, CDIF1296T_03072), anaerobic sulfite reductase subunit (*asrA*, *asrB*), serine acetyltransferase (*cysA*), and O-acetylserine sulfhydrylase (*cysM*) were downregulated 1.4- to 4.6-fold. Interestingly, all 8 genes encoding ATP synthase subunits of photosynthesis in CDL cells were upregulated (1.7-2.6-fold), whereas only 4 genes were upregulated in CDH cells. Overall, genes involved in the V-type ATP synthase of oxidative phosphorylation and photosynthesis pathways exhibited a more pronounced downregulation in CDL cells than in CDH cells, while genes associated with ATP synthase subunits were significantly upregulated in CDL cells. Most DEGs of membrane transport in CDL and CDH cells belonged to the phosphotransferase system (PTS), ABC transporters and bacterial secretion system. 45 genes encoding PTS system transporter subunits and PTS system galactitol-specific transporter subunits in CDL cells were downregulated 1.1- to 7.5-fold, while in CDH cells, 42 genes associated with PTS system transporter subunits were downregulated 1.0- to 8.4-fold. Similar results were also found in ABC transporters in both CDL and CDH cells. These results indicated that the activities of gene clusters involved in the PTS and ABC transporters were weakened significantly in CDL and CDH cells, especially in CDH cells.

Quorum sensing, signal transduction, biofilm formation, and flagellar assembly are the core parts of the pathogenic activity of *C. difficile*. Quorum sensing is an important part of infection induced by CD. When the density of bacteria reaches a critical value, signal molecules (AIs) are generated immediately and further induce quorum sensing, accompanied by the formation of biofilms and corresponding secretions (toxin proteins A and B). KEGG results showed that most DEGs in CDL and CDH cells were related to quorum sensing and biofilm formation, and a significant downregulation trend of gene expression associated with biofilm formation was observed in CDL and CDH cells (Figure 7 and Supplementary Table S4). With regard to quorum sensing, there were 29 DEGs (10 down- and 19 upregulated) in CDL cells, including the expression levels of *tcdA* and *spo0A*, which were downregulated by 1.66-fold and 1.09-fold, respectively. In addition, genes encoding oligopeptide ABC transporter and amino acid permease family protein were individually downregulated 1.3- to 3.6-fold and 4.2-fold. The expression levels of genes encoding preprotein translocase, lantibiotic ABC transporter ATP-binding protein, lantibiotic ABC transporter permease, and lantibiotic resistance two-component response regulator were upregulated 1.1- to 3.4-fold in CDL cells, and genes encoding signal peptidase I were upregulated 1.3- to 1.6-fold. In addition, *luxS* associated with S-ribosylhomocysteinase in CDL cells was upregulated 1.1-fold, and *oxaA1* encoding sporulation membrane protein was upregulated 2.1-fold. In CDH cells, there were a total of 19 DEGs (10 down- and 9 upregulated), including genes encoding oligopeptide ABC transporter permease, oligopeptide ABC transporter ATP-binding protein, and oligopeptide ABC transporter substrate-binding protein, which were downregulated by 1.3- to 2.0-fold, whereas the expression levels of *tcdA* and *tcdB* were increased by 2.6-fold and 3.7-fold, respectively, and *spo0A* was upregulated 1.9-fold. Moreover, genes associated with preprotein translocase (1.0- to 3.8-fold upregulated), EamA-like transporter family protein (CDIF1296T_01483, 3.8-fold upregulated), and riboflavin biosynthesis protein (*ribD*, 3.4-fold upregulated) in CDH cells were also upregulated. In signal transduction, 39 DEGs were associated with two-component systems in CDL cells, including 13 downregulated and 26 upregulated genes. The downregulated genes included a negative regulator of flagellin synthesis (anti-sigma-d factor) and flagellin subunits (4.1- and 4.7-fold downregulated, respectively), acetyl-CoA acetyltransferase (*thlA1* and *thlA2*, 4.5- and 5.6-fold downregulated, respectively), and butyrate–acetoacetate CoA-transferase subunit (*ctfA* and *ctfB*, 5.3- and 5.9-fold downregulated, respectively). For the upregulated genes, there were lantibiotic ABC transporter permease (1.4- to 2.6-fold upregulated), potassium-transporting ATPase subunit (1.3- to 1.7-fold upregulated), D-alanine—poly (phosphoribitol) ligase subunit and D-alanyl transferase (1.3- to 2.2-fold upregulated). For CDH cells, 21 DEGs were associated with two-component systems, including 14 downregulated and 7 upregulated genes. Specifically, the downregulated genes included butyrate—acetoacetate CoA-transferase subunit (3.6- to 5.1-fold downregulated), acetyl-CoA acetyltransferase (4.9-fold downregualted), and chemotaxis protein (1.7-fold downregulated), while the upregulated genes contained ABC transporter permease (2.5-fold upregulated). In biofilm formation, downregulated genes in CDL cells included negative regulator of flagellin synthesis (*flgM*, 4.1-fold downregulated), glucose-1-phosphate adenylyltransferase, glycogen biosynthesis protein, glycogen synthase, glycogen phosphorylase (4.2- to 4.8-fold downregulated), and PTS system glucose-specific transporter subunit IIA (7.3-fold downregulated). For CDH cells, downregulated genes included glucose-1-phosphate adenylyltransferase, glycogen biosynthesis protein, glycogen synthase, glycogen phosphorylase (1.9- to 2.2-fold downregulated), and PTS system glucose-specific transporter subunit IIA (7.6-fold downregulated). Flagella and chemotaxis contribute not only to the motility of *C. difficile* and to obtaining nutrients, but also to intestinal tissue infection. The activity of gene clusters associated with flagellar assembly and bacterial chemotaxis in CD weakened, especially in CDH cells. In CDL cells, 9 genes associated with flagellar motor switch protein, flagellar protein, negative regulator of flagellin synthesis (anti-sigma-d factor), flagellar hook-associated protein, and flagellar cap protein were downregulated by 1.2- to 4.7-fold, whereas 2 genes associated with chemotaxis protein were upregulated by 1.6- to 1.9-fold. For CDH cells, 6 genes encoding flagellar basal-body rod protein, flagellar hook-length control protein, flagellar hook protein, chemotaxis protein, flagellar biosynthesis protein FliP, flagellar biosynthesis protein FlhA, and flagellar basal body rod protein FlgG were downregulated by 1.0- to 1.2-fold, and 5 genes encoding chemotaxis protein were downregulated by 1.0- to 2.0-fold. Overall, the expression levels of *tcdA* and *spo0A* were reduced in CDL cells, whereas these two were enhanced in CDH cells. *luxS* and relevant genes encoding signal peptidase I in CDL cells were upregulated, while this trend did not appear in CDH cells. Genes associated with inhibiting flagellum synthesis protein during signal transduction in CDL cells were significantly downregulated; however, this phenomenon was not found in CDH cells. CDL cells had an improved ability to suppress biofilm formation and flagellar assembly, causing an enhancement in bacterial chemotaxis. Interestingly, these trends were reversed in CDH cells.

Drug resistance (antimicrobial) consisted of mainly vancomycin resistance, cationic antimicrobial peptide (CAMP) resistance and beta-lactam resistance (Supplementary Table S4). Sixteen upregulated genes in CDL cells included vancomycin resistance (7, 1.1- to 2.2-fold), CAMP resistance (6, 1.3- to 2.2-fold), and penicillin-binding protein of beta-lactam resistance (3, 1.6- to 3.9-fold). In CDH cells, 9 upregulated genes included vancomycin resistance (4, 1.1- to 2.3-fold), CAMP resistance (3, 2.0- to 3.1-fold), and penicillin-binding protein (2, 1.0- to 2.9-fold). Overall, drug resistance metabolism was more active in CDL cells than in CDH cells, and the expression levels of relevant drug resistance genes were significantly increased.

#### Differences Between CDL and CDH Cells

The top 20 KEGG pathways in CDL and CDH cells are showed in Figure 7 and Supplementary Table S4. The shared pathways in CDL and CDH cells were ribosome; fructose and mannose metabolism; valine, leucine and isoleucine biosynthesis; C5-branched dibasic acid metabolism; peptidoglycan biosynthesis; PTS; and oxidative phosphorylation. These pathways were focused mainly on the aspects of energy transport, accompanied by oxidative phosphorylation of specific carbon and nitrogen sources to provide energy. With regard to the different pathways, carbon, nitrogen source and energy metabolism were still the main pathways (butanoate metabolism, methane metabolism, photosynthesis, and arginine biosynthesis) in CDL cells, whereas the metabolism of cofactors and vitamins (thiamine metabolism, pantothenate and CoA biosynthesis, and riboflavin metabolism) and drug resistance (antimicrobial) were the main pathways in CDH cells. The top 20 upregulated and downregulated genes with the greatest changes in CDL and CDH cells are showen in Supplementary Table S2. Compared with CDC cells, the greatest change among upregulated genes of CDL cells was that in CDIF1296T_01114 (6.25-fold upregulated), which encodes transcriptional regulator synthesis; in addition, genes associated with enzymes during the metabolism of carbon and nitrogen sources were upregulated by 5-fold. For CDH cells, the greatest change among upregulated genes was that in CDIF1296T_01782 (6.19 fold upregulated), which encodes riboflavin synthase subunit beta. These results were consistent with the top 20 KEGG pathways. Notably, one-third of the top 20 upregulated genes in CDH cells were related to sporulation proteins, e.g., stage II sporulation protein e (*spoIIE*, 5.83-fold upregulated), stage III sporulation-like protein (*spoIIAE*, 4.97-fold upregulated), stage II sporulation protein P (*spoIIP*, 4.82-fold upregulated), germination protease (*gpr*, 4.79-fold upregulated), and stage III sporulation protein AC (*spoIIAC*, 4.22-fold upregulated). For CDL cells, the top 20 upregulated genes were concentrated mainly in carbon, nitrogen and energy metabolism. The top 20 downregulated genes in CDL cells included PTS system transporter (5.65- to 7.51-fold downregulated), V-type ATP synthase (5.96- to 6.67-fold downregulated), phosphosugar isomerase (6.83- to 7.15-fold downregulated), and bifunctional protein (6.29-fold downregulated). For CDH cells, the top 20 downregulated genes were associated with bifunctional protein (9.62-fold downregulated), and PTS system transporter (4.85- to 8.37-fold downregulated). Overall, the top 20 downregulated genes of CDL and CDH cells were focused mainly on bifunctional protein, PTS system transporter and V-type ATP synthase.

## Discussion

Infection triggered by *C. difficile* causes serious harm to human health, and this incidence trend is spreading globally. Metronidazole and vancomycin, as the main representative of antibiotics, are still the first choice in the treatment of CDI despite their increasing drawbacks, such as the emergence of multidrug-resistant bacteria and the destruction of the intestinal immune system and microflora (Smits et al., 2016). Therefore, it is urgent to find new therapies to overcome these problems. Among a host of new therapies, probiotics and fecal microbiota transplantation (FMT) have gained popularity and have been widely used in clinical CDI treatment (Yang and Yang, 2019b). Essentially, FMT is used mainly in the treatment of severe CDI (rCDI) and has produced a remarkable therapeutic effect (Juul et al., 2018); however, there are no uniform operating codes and standards, especially in the treatment of pediatric patients (Chen et al., 2017). Moreover, potential pathogenic factors may exist in donor feces. Probiotics or probiotics in combination with antibiotics are widely used in the treatment of mild and moderate CDI, and an increasing number of clinical reports have indicated that these probiotic therapies exert a satisfactory effect with very few side effects, suggesting that traditional antibiotics are likely to be replaced by probiotics (Mills et al., 2018). It should be noted that there are still some problems to be resolved and worth discussing about probiotic therapy, and one of them is the dosage (Shen et al., 2017). Physicians generally believe that the higher the dosage of probiotics is, the better the therapeutic effect, but there is no theoretical basis to support this traditional concept. Whether the efficacy of probiotics against different infections caused by different pathogenic bacteria presents a dose-response relationship is still unknown. In addition, a few reports indicated that an overdose of probiotics might interfere with the normal gut microflora (Langella and Chatel, 2019). Thus, it is necessary to determine the precise dosages of probiotics in clinical treatment. Currently, most studies on the antibacterial activity of probiotics against *C. difficile* have not provided a substantial explanation of the antibacterial mechanism, especially the effects of different dosages of probiotics against *C. difficile* at the transcriptional level. Thus, we used several physiological indexes in combination with a transcriptome analysis to explore the changes in the CD response to YH68 at different doses, especially the different changes between CD cells triggered by low and high doses of YH68 at the transcriptional level.

Gao et al., 2010 found that the incidence of CDI was reduced significantly after the ingestion of probiotic capsules (*L. acidophilus* CL1285^®^ + *L. casei LBC80R*^®^ Bio-K + CL1285); moreover, the therapeutic effect of 1 × 10^9^ CFU of probiotics is better than that of 0.5 × 10^9^ CFU. Szajewska et al., 2019 investigated the usage of *L. rhamnosus* GG (LGG) in the treatment of acute gastroenteritis in children and found that a dosage of more than 10^10^ CFU of LGG daily shortened the time of diarrhea, but the actual therapeutic effect was not significant. This result suggested that not all probiotics at high dosages exert an outstanding therapeutic effect. In this study, the growth, cell membrane integrity, toxin production, and the expression levels of the *tcdA/tcdB* genes in CD exhibited a dose-suppressive response with increasing doses of YH68. Interestingly, the preventive inhibitory effect of YH68 against CD is stronger than that of therapeutic inhibition, suggesting that YH68 could defend against the invasion of CD. This result implies that the ingestion of a certain dosage of probiotics at an early time can theoretically greatly reduce the probability of infection induced by *C. difficile*. This approach could be an effective precaution for medical workers and inpatients to defeat the first invasion of *C. difficile*.

According to the physiological test results, high and low doses of YH68-CFCS were further selected to treat CD cells (CDH and CDL) within a short time to explore how these two doses affect CD cells at the transcriptional level. Compared with those in the control cells CDC, 1758 and 1166 DEGs were found in CDL and CDH cells, respectively. The vast majority of DEGs were focused mainly on carbohydrate, amino acid, energy and transmembrane transport metabolic pathways, especially the first two (Figure 7). *C. difficile* changes its metabolic pathways based on the different carbon and nitrogen sources and subsequently generated energy through redox of these nutrient substances to support their normal growth (Hofmann et al., 2018). Both CDL and CDH cells exerted high activity in carbohydrate metabolism, and the largest difference between CDL and CDH cells was butanoate metabolism. All genes associated with butanoate metabolism in CDL cells were downregulated, suggesting that this metabolic pathway was suppressed dramatically by the low dose of YH68-CFCS. Amino acid metabolism plays another critical role in the growth of *C. difficile* (Fletcher et al., 2018), and changes in significant pathways in CDL and CDH cells were almost the same, e.g., proline and valine are important nutrients for *C. difficile*. Isoleucine and valine provide reduction for proline reductase during the Stickland reaction to promote the overall energy supply system in *C. difficile* (Hofmann et al., 2018). Notably, the biggest change of the carbohydrate and amino acid metabolic pathways in CDL and CDH cells might not just due to the YH68-CFCS, but also depended on the culture environment of CD cells. Some nutrient substances in the BHI and MRSC broths were also important for CD cells, such as glucose and peptone. Interestingly, the carbohydrate and amino acid metabolic activities in CDL were more active than that in CDH, though the volume and concentration of BHI broths in CDL and CDH were same. This phenomenon was most likely due to some substances secreted in YH68-CFCS reached a high level in CDH and these substances inhibited the metabolic activities of CD cells, whereas these substances in CDL was low, thus had almost no effects on CD cells. Highly active pathways during energy metabolism in CDL and CDH cells were almost the same. Membrane transport is closely related to nutrient metabolism. CDL and CDH cells possessed the same top three pathways: the PTS, ABC transporters and bacterial secretion system. Most genes associated with the PTS were downregulated at both doses, especially in CDH cells. Changes at the transcriptome level of CD cells revealed that the main metabolic pathways involved in carbon and nitrogen source metabolism in CDL and CDH cells changed substantially in comparison with those in CDC. In addition, the expression levels of genes involved in energy and membrane transport were suppressed by high and low doses of YH68-CFCS, suggesting that these two doses interfered with not only the way CD obtains energy but also the process of transporting nutrients.

With regard to the pathogenic aspects of CD, there were also large differences between CDL and CDH cells, including in quorum sensing, signal transduction, biofilm formation, and flagellar assembly. The expression levels of *tcdA* and *spo0A* related to quorum sensing increased significantly in CDH cells at 3 h, whereas this trend was the opposite in CDL cells. The result of this part was inconsistent with that of the initial physiological experiment was mainly due to the different proportions of YH68-CFCS and different concentrations of CD cell suspension used in these experiments. More importantly, the processing time was different, including 24 and 48 h in the physiological experiment and 3 h in the transcriptome experiment. During the transcriptome experiment, CD cells continued to grow for a short time because there were relatively adequate nutrients in BHI broths at the beginning, and thus the expression levels of *tcdA* and *spo0A* increased temporarily. Previous studies revealed that some antibiotics stimulated the expression levels of *tcdA/B* and *spo0A*, subsequently inducing additional toxin and spore production, which are accompanied by severe side effects (Zarandi et al., 2017; Guh and Kutty, 2018; Yang and Yang, 2018). This feature CDH cells is analogous to that induced by some antibiotics, suggesting that CDH cells have the potential to induce additional toxin and spore production during the clinical treatment of CDI. The expression levels of genes associated with *luxS* and signal peptidase I in CDL cells were upregulated, but this trend did not appear in CDH cells, suggesting that CDH cells can block and silence the expression of key genes in quorum sensing. Genes associated with biofilm formation in both CDL and CDH cells exhibited a downregulation trend. For flagellar assembly and motility, their corresponding genes in CDL cells were inactive and inhibited, such as the expression levels of *fliN_1*, *flgM*, *flgK*, *flgL*, *fliS1*, *fliS2*, *fliD*, *fliC*, and *flgE*, which were significantly downregulated; this trend was positively correlated with the expression level of *tcdA* in CDL cells. Previous studies demonstrated that the transcriptional level of virulence genes was influenced by the activity of flagellum synthesis (Aubry et al., 2012). Therefore, the production of toxin decreased presumably due to the suppressed expression levels of genes associated with the flagellum in CD. The expression level of genes associated with bacterial chemotaxis was enhanced in CDL cells but not in CDH cells, suggesting that this change might be due to the concentration of nutrients and harmful substances in the environment (Hofmann et al., 2018). Genes encoding drug resistance exerted a high expression level in CDL cells, suggesting that the low dose of YH68 might lead to enhanced drug resistance in *C. difficile* and further induce the development of multidrug-resistant strains. From the perspective of clinical application, high and low doses of YH68 might have a good therapeutic effect in the treatment of CDI. From further analysis, a high dose (CDH) seems to be more appropriate than a low dose for the middle and later stages of the disease because there are many more *C. difficile* vegetative cells and spores in the infected tissues (rCDI) at this time, and their quorum sensing system could be blocked by CDH. A low dose (CDL) seems to be more appropriate for preinfection treatment (primary or mild CDI) because the activity of flagellar assembly of *C. difficile* could be weakened, as well as reduced expression level of genes encoding toxin and spores production, finally further preventing the invasion and colonization of *C. difficile* strains. The top 20 genes with the greatest differences in expression levels (up- and downregulation) showed that the activities of energy metabolism and transport at both doses were weakened significantly. In addition, one-third of the upregulated genes in CDH cells were related to spore germination, suggesting that a high dose of YH68 might be a potential germination agent for specific *C. difficile* strains that can stimulate the production of spores.

The overwhelming majority of probiotics and probiotics in combination with antibiotics used in the treatment of CDI has shown an outstanding therapeutic effect, except for a few ineffective cases (Goldenberg et al., 2018). The suitable dosage of probiotics has always been a vague question in clinical treatment. Physicians preferred to use the high dosage to achieve the best therapeutic effect, although with a few ineffectual and even adverse reactions. The results from this study indicated that the different dosages of probiotics might cause various side effects, e.g., the high dose of YH68 induced *C. difficile* to produce additional toxin, whereas the low dose stimulated the drug resistance of *C. difficile*. This finding may be one reason for the failure of probiotics used in clinical cases. Furthermore, the results in this study revealed that a higher dosage of probiotics does not necessarily lead to better therapeutic effects. Admittedly, the actual therapeutic effects of probiotics in clinical treatment are determined not only by the dosage of probiotics, but also by other factors, such as human gut cells and immune system (Yang and Yang, 2019b). Actually, there is a complex interaction between probiotics, human gut cells and immune system. The findings in this study only revealed effects of probiotics at different doses against *C. difficile in vitro*. Therefore, whether the therapeutic effects of probiotics have a similar dose-response relationship in clinic needs a further clinical research to verify.

Theoretically, if the main pathogenic strain that causes a disease can be determined first, then the precise dosage of probiotics can be chosen and delivered. For example, if *C. difficile* ATCC 9689 is the main pathogenic strain in the patient’s stool, then the CDH strategy should be the suitable dose to use because of the low spore-producing capacity of *C. difficile* ATCC 9689 (Garneau et al., 2014). Meanwhile, if *C. difficile* ATCC 43255 is the pathogen, then the CDL strategy should be the suitable dosage because of the high spore- and toxin-producing capacity of *C. difficile* ATCC 43255 (Garneau et al., 2014). The inhibitory effect of different probiotics against different *C. difficile* strains may not occur via a non-dose-response relationship. Thus, the precision dosage of different probiotics used in the treatment of specific diseases should be determined to meet the demand of precision medicine.

## Conclusion

In this study, *C. difficile* ATCC 9689 (CD) was exposed to different doses of YH68-CFCS or YH68 cell suspension. A series of physiological indexes indicated that CD was suppressed significantly at the physiological level as the dose of YH68 increased. Transcriptional analysis showed that the expression levels of genes associated with carbohydrate, amino acid, energy metabolism and membrane transport in CD cells were suppressed by both high and low doses of YH68-CFCS. However, there were some differences between the two doses, reflecting in the different change in activities in pathogenic pathways (quorum sensing, signal transduction, flagellar assembly, biofilm formation, and drug resistance) of CD. This study provides some implications that the precise dosages of probiotics and their metabolites should be determined in advance before they used in the clinical prevention or treatment of CDI. Precision dosage has the potential to reduce and avoid some potential risk factors, which would align well with the target of achieving the best therapeutic effect with minimal side effects.

## Data Availability Statement

The datasets generated for this study can be found in the NCBI under accession number PRJNA597573.

## Author Contributions

JY and HY designed experiments. JY performed the experiments. JY and HY analyzed the data and wrote the manuscript. Both authors read and approved the final manuscript.

## Conflict of Interest

The authors declare that the research was conducted in the absence of any commercial or financial relationships that could be construed as a potential conflict of interest.

## Footnotes

1 http://bowtie-bio.sourceforge.net/index.shtml

2 https://htseq.readthedocs.io/en/master/
