# Supplementary material for: Transcriptome Analysis of the Clostridioides difficile Response to Different Doses of Bifidobacterium breve
Source: Front Microbiol. 2020 Jul 31;11:1863. doi: 10.3389/fmicb.2020.01863 (PMC7411088; doi:10.3389/fmicb.2020.01863)
Supplement: Supplementary file 2 [file Data_Sheet_1.PDF]

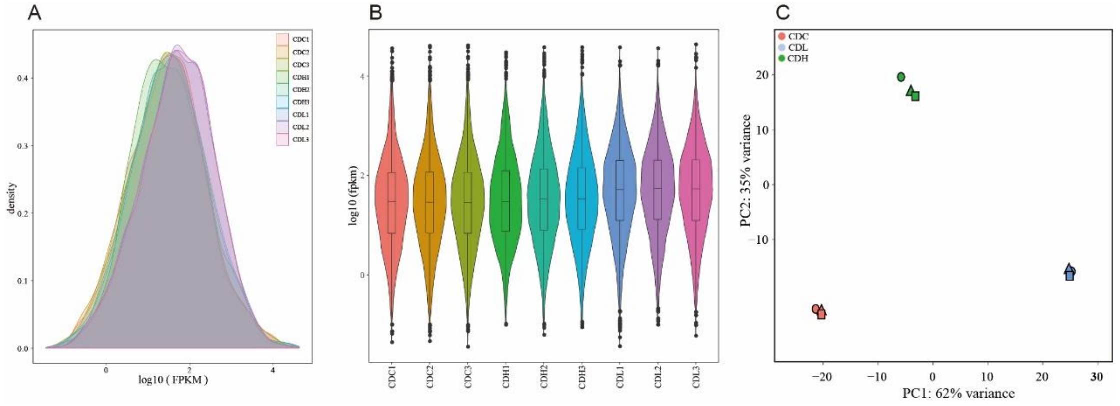

Figure S1. Gene expression model of *C. difficile*. (A) FPKM density, (B) density distribution, and (C) principal component analysis.

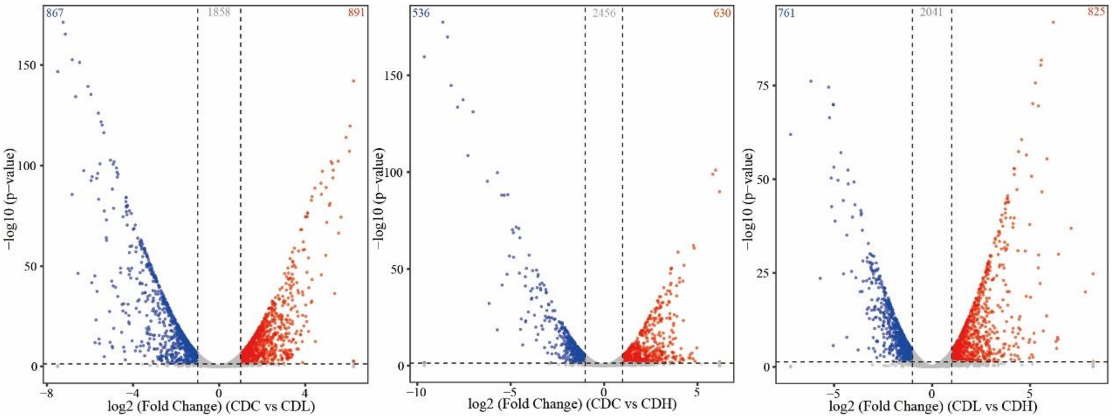

Figure S2. Volcano plot highlighting genes whose transcript levels changed by greater than 2-fold and met the significance threshold P-value≤0.05. Red points represent genes with increased transcript levels, while those highlighted in blue had decreased levels. Gray points represent genes whose results failed to meet the significance threshold.
